# Phytochemical, Antioxidant, Anti-Inflammatory, and Thrombolytic Properties of *Cleisomeria lanatum* (Lindl.) Lindl. ex G. Don

**DOI:** 10.1155/2022/5660527

**Published:** 2022-05-26

**Authors:** Minhajur Rahman, Abu Taleb Surag, Roxy Begum, Md. Shakhuat Hossain Tusher, Mohammed Kamrul Huda

**Affiliations:** ^1^Department of Botany, University of Chittagong, Chittagong, Bangladesh; ^2^Ecology and Phytochemistry Lab, Department of Botany, University of Chittagong, Chittagong, Bangladesh; ^3^In Silico Drug Design Lab, Department of Botany, University of Chittagong, Chittagong, Bangladesh; ^4^Department of Pharmacy, Southern University, Chittagong, Bangladesh

## Abstract

For the first time, *Cleisomeria lanatum* (family: Orchidaceae) has been investigated for its phytochemical, antioxidant, anti-inflammatory, and thrombolytic properties. All phytochemicals studied were identified in varying degrees during qualitative screening. In quantitative screening, a maximum of 106.02 ± 0.08 mg/g alkaloids (root), 179.67 ± 8.83 mg/g phenols (stem), 17.34 ± 0.88 mg/g flavonoids (stem), 73.67 ± 1.76 mg/g tannins (stem), and 180.04 ± 0.02 *μ*g/mL proteins (root) were detected. Antioxidant, anti-inflammatory, and thrombolytic properties were investigated using a free-radical scavenging assay, heat-induced albumin denaturation assay, and blood clotting inhibition assay. The root extracts had the most effective antioxidant (IC_50_ = 67.98 *μ*g/mL) and anti-inflammatory (IC_50_ = 60.86 *μ*g/mL) properties, whereas the stem extracts had the most effective thrombolytic property (IC_50_ = 163.8 *μ*g/mL). The bioactivities studied also had a significant positive relationship (*r* = 0.9; *p*  <  0.05) with the amount of phenolics and tannins.

## 1. Introduction

Orchids are the most numerous and diversified species of flowering plants, which were originally grown for their lovely flowers [1]. Although orchids are well known for their horticultural and commercial value, their therapeutic potential has yet to be fully investigated [2]. According to current research, the Orchidaceae family is home to a plethora of therapeutic plants [3].

Orchids have long been prized for their medicinal properties and as a source of pharmaceuticals [4, 5]. Arundin, dendroflorin, moscatilin, and vanillin are potent anticancer, antitumor, and anti-inflammatory agents discovered from orchids [6]. They have also been appreciated for their therapeutic quality and as a source of medications for a long time [4]. Since time immemorial, certain orchid species have been employed as traditional cures for a range of ailments [7, 8]. Despite the fact that they have long been used as traditional medicines, their medicinal potential is yet to be explored [5]. Moreover, orchids have been used to treat a variety of ailments and diseases, including chest pain, arthritis, syphilis, jaundice, cholera, acidity, eczema, tumor, piles, tuberculosis, wounds, stomach disorders, boils, inflammation, menstrual disorders, spermatorrhea, leucoderma, slantendicular, muscular pain, earache, sexually transmitted diseases, blood dysentery, hepatitis, bone fractures, rheumatism, asthma, malaria, paralysis, and dyspepsia [4]. Therefore, some potent anticancer, antitumor, and anti-inflammatory agents, i.e., arundin, dendroflorin, moscatilin, and vanillin, are also discovered from orchids [6]. Genus *Cleisomeria* has ethnomedicinal potential as well as bioactive properties [5]. The studied orchid genus has been recorded to be used in ethnomedicine for diarrhoea, aphrodisiac, chronic constipation, and bone fractures [9]. Bioactivities of species belonging to the same genus are extremely similar; according to the report, when one species in a genus is identified to have medicinal characteristics, other species in that genus are also determined to have medicinal properties [10]. According to the literature review [11, 12], the phytochemical and biological features of *Cleisomeria lanatum* are still unknown. Therefore, in light of the foregoing discussion, *C. lanatum,* a promising member of the *Cleisomeria* genus, was chosen to be studied with its phytochemical screening and bioactivity (antioxidant, anti-inflammatory, and thrombolytic) analysis.

## 2. Materials and Methods

### 2.1. Plant Material Collection

Plants were collected from Cox's Bazar, Chittagong, Bangladesh. The collected plants were carefully examined and authenticated by Professor Dr Muhammed Kamrul Huda, Department of Botany, University of Chittagong. The fresh, disease-free plants were selected for experiments. The leaves, root, and stems of the studied orchid samples were used for the qualitative and quantitative estimation of secondary metabolites and their bioactivity tests.

### 2.2. Preparation of the Plant Extract

For further analysis, 50 g of powdered samples from each part was taken. In a conical flask, 100 ml of methanol was added to 50 g of the sample. Then, it was shaken very well for 30 minutes and kept overnight; after that, it was shaken again and sonicated for 10 minutes and filtered using the Whatman No.1 filter paper.

### 2.3. Phytochemical Analysis

#### 2.3.1. Qualitative

Following established methods, alkaloids [13], flavonoids [14], saponins [15], tannins [16], phenols [17], terpenoids [18], steroids [18], glycosides [16], cardiac glycosides [19], quinine [20], coumarins [20], proteins [21], and resins [19] were qualitatively examined.

#### 2.3.2. Quantitative

Standard methods were also used to quantify alkaloids [16], phenols [22], tannins [23], flavonoids [24], and proteins [25].

### 2.4. Bioactivity

#### 2.4.1. Antioxidant Activity

The antioxidant activities of the methanolic crude extracts of the leaves, stems, and roots of *C. lanatum* and the standard antioxidant ascorbic acid were assessed on the basis of the free-radical scavenging effect of the 1, 1-diphenyl1-picrylhydrazyl (DPPH) free-radical activity in accordance with the method described by the standard method with a slight modification [26]. The absorbance of DPPH solution (control solution “A”) was measured at 517 nm using a UV-visible spectrophotometer. Ascorbic acid served as a positive control. Lower absorbance of the reaction mixture indicated a higher free-radical scavenging activity. The scavenging activity against DPPH was calculated using the following equation:

(1) $$\begin{array}{c} \text{Scavenging activity}\left(\%\right)=\left(\frac{A-B}{A}\right)\times 100, \end{array}$$

where *A* represents the absorbance of the control (DPPH solution without the sample) and *B* represents the absorbance of the DPPH solution in the presence of the sample (extract/ascorbic acid).

#### 2.4.2. Anti-Inflammatory Activity

The anti-inflammatory activity of *C. lanatum* was studied by using inhibition of the albumin denaturation technique, which was studied according to the standard method [27, 28]. The reaction mixture consists of test extract (50, 100, 150, 200, 250, and 300 *μ*g/mL) concentrations and 5% aqueous solution of egg albumin, and the pH (5.6 ± 0.2) of all reaction mixtures was adjusted by 1N HCl. The sample extracts were incubated at 37°C for 20 min and then heated to 51°C for 20 min, and after cooling the samples, the turbidity was measured at 660 nm using the spectrophotometer. The experiment was performed in triplicate [29].

#### 2.4.3. Thrombolytic Activity

The experiment for clot lysis was carried out as per the standard method [30] with a slight modification. In this method, venous blood drawn from healthy volunteers was transferred into a different preweighted sterile Eppendorf tube (500 *μ*L/tube) and incubated at 37°C for 45 minutes. After clot formation, serum was completely removed (aspirated out without disturbing the clot formed). Each tube having a clot was again weighed to determine the clot weight. Each Eppendorf tube containing the clot was properly labelled, and 100 *μ*L of the plant extract was added to the tubes. All tubes were then incubated at 37°C for 90 minutes and observed for clot lysis. After incubation, the fluid obtained was removed and the tubes were again weighed to observe the difference in weight after clot disruption. The difference obtained in weight taken before and after clot lysis was expressed as the percentage of clot lysis. Streptokinase and water were used as positive and negative (nonthrombolytic) controls, respectively. The experiments were repeated several times with the blood samples of different volunteers.

### 2.5. Statistical Analysis

All experiments were performed in triplicate. The obtained data were analyzed using Microsoft Office Excel 2010 for statistical analysis. Pearson's correlation coefficient analysis was used to find the correlation between quantitative phytochemicals with the bioactivities previously mentioned .

## 3. Results and Discussion

### 3.1. Phytochemical Analysis

#### 3.1.1. Qualitative

Qualitative screening revealed the presence of alkaloids, flavonoids, saponins, tannins, phenols, terpenoids, steroids, glycosides, cardiac glycosides, quinines, coumarins, proteins, and resins in varying degrees, as given in Table 1. The current study agrees with the work on phytochemical studies [31] and other relevant studies on ten orchids [3].

#### 3.1.2. Quantitative

*(1) Total alkaloid*. The total alkaloid content of *C. lanatum* leaf, stem, and root methanolic extracts recorded was 98.23 ± 0.22, 101.78 ± 1.15, and 106.02 ± 0.08 mg/g, respectively. In comparison to leaf and stem extracts, the root extract had the most alkaloids (106.02 ± 0.08 mg/g).

*(2) Total flavonoid*. Methanolic extracts of *C. lanatum* leaf, stem, and root produced 3.2 ± 0.3, 17.34 ± 0.88, and 0.45 ± 0.15 mg flavonoids per gram, respectively. In comparison to leaf and root extracts, stem extracts contained the most flavonoids (17.34 ± 0.88 mg/g).

*(3) Total phenol*. The total phenolic content in the methanolic crude extracts of the leaf, stem, and roots of *C. lanatum* was estimated using the Folin Ciocalteu's reagent (FCR) and compared with the gallic acid equivalent standard curve equation *y* = mx + *c* (*y* = 0.0039*x* + 0.0388, *R*^2^ = 0.9976). In comparison to leaf and root extracts, stem extracts contained the most phenols 179.67 ± 8.83 mg/g.

*(4) Total protein*. The results of the total protein contents of leaf, stem, and root methanolic crude extracts of *C. lanatum* were found as 133.37 ± 0.75, 84.20 ± 0.23, and 180.04 ± 0.02 *μ*g/mL, respectively. The highest amount of protein was found in root extracts 180.04 ± 0.02 *μ*g/mL, equivalent to bovine serum albumin, in comparison with leaf and stem.

*(5) Total tannin*. In this study, tannic acid was used as the standard, and the total tannin content was expressed as tannic acid equivalents (TAEs). Absorbance was measured at 700 nm. From the quantitative estimation of tannins, the results of total tannin contents of leaf, stem, and root methanolic crude extracts of *C. lanatum* were found as 40.33 ± 1.33, 73.67 ± 1.76, and 52.33 ± 11.31 mg g^−1^, respectively. The highest amount of tannin found in the stem extract was 73.67 ± 1.76 mg/g, in comparison with leaves and roots.

**Figure 1:**
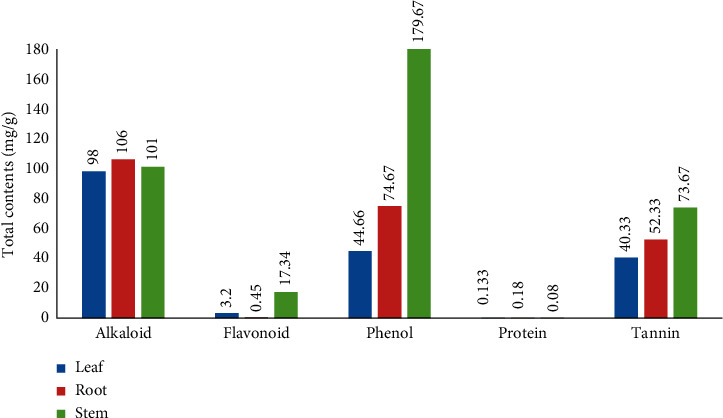
Quantitative phytochemical analysis of *C. lanatum*.

The overall result of the quantitative phytochemical study of *C. lanatum* is shwon in Figure 1. The current study agrees with the work on orchids for alkaloids, flavonoids, phenols, proteins, and tannins, respectively [32–34].

### 3.2. In Vitro Bioactivity

#### 3.2.1. Antioxidant Activity

**Figure 2:**
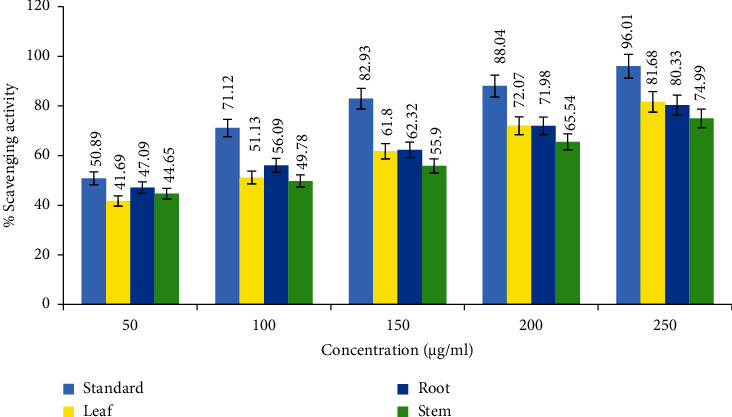
Antioxidant activity of leaves, root, and stems of *C. lanatum* with the standard (ascorbic acid).

**Figure 3:**
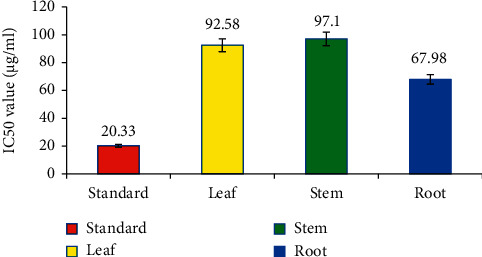
Antioxidant activity of *C. lanatum* with IC_50_ values.

The antioxidant activity of leaves, stem, and roots of *C. lanatum* was tested by DPPH-scavenging activity. The percentage of scavenging activity was subjected to regression and correlation to establish the IC_50_ value (half the maximum inhibitory concentration). A free-radical scavenging assay was used to assess the antioxidant activity of leaf, stem, and root extracts with IC_50_ values of 92.58, 97.10, and 67.98 *μ*g/mL, respectively, have the highest antioxidant activity when compared to the standard (20.33 *μ*g/mL) (Figures 2 and 3). The current findings are consistent with the work on *Trudelia cristata* and *Gastrochilus acutifolius*, where the highest DPPH-scavenging activity was assessed as IC_50_ values 69 *μ*g/mL and 341.79 *μ*g/mL, respectively [35].

#### 3.2.2. Anti-Inflammatory Activity

**Figure 4:**
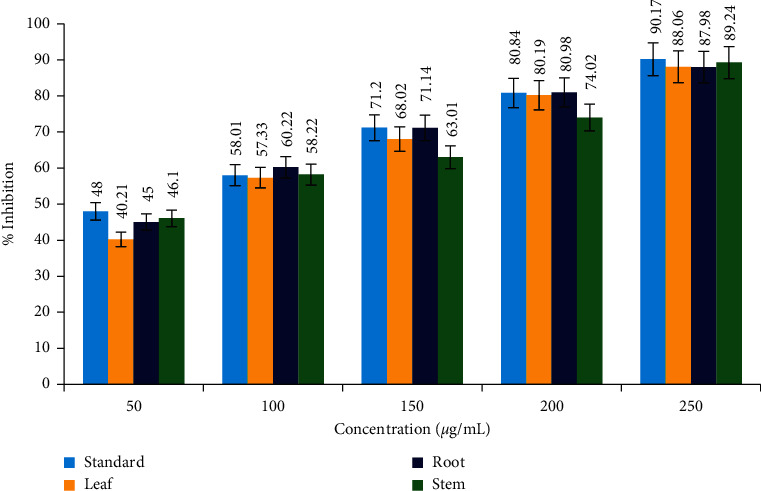
Anti-inflammatory activity of *C. lanatum* with the standard (acetyl salicylic acid).

**Figure 5:**
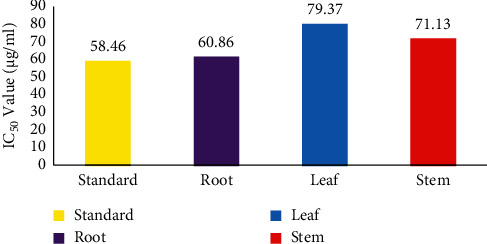
Anti-inflammatory assay of *C. lanatum* with IC_50_ values.

As part of the anti-inflammatory investigation, the protein denaturation ability of leaves, bulbs, and roots of *C. lanatum* was studied (Figures 4 and 5). The percentage of protein denaturation ability was subjected to regression and correlation to establish the IC_50_ value (half the maximum inhibitory concentration). The anti-inflammatory efficacy was determined using the heat-induced albumin denaturation assay. The leaf, stem, and root extracts had IC_50_ values of 79.37, 71.13, and 60.86 *μ*g/mL, respectively, with the root extract having the best anti-inflammatory effect when compared to the standard (58.46 *μ*g/mL).

The current study is consistent with the work on the anti-inflammatory activity of *Dendrobium macrostachyum*, with IC_50_ values of 114.13 and 135.818 *μ*g/mL, respectively [36]. This lends credence to the current investigation.

#### 3.2.3. Thrombolytic Activity

**Figure 6:**
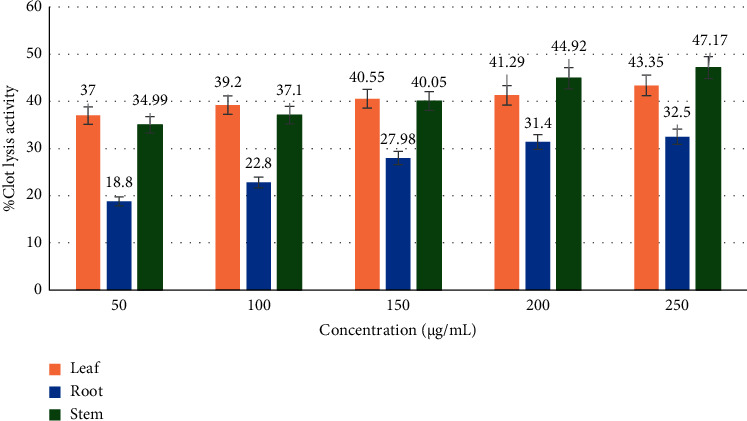
Thrombolytic activity of *C. lanatum*.

**Figure 7:**
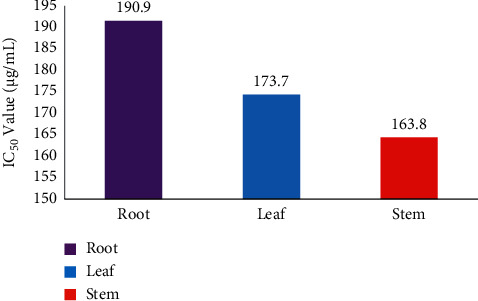
Thrombolytic activity of *C. lanatum* with IC_50_ values.

To discover the thrombolytic activity of *C. lanatum*, leaves, roots, and stems were assessed with the clot lysis activity (Figures 6 and 7). The percentage of the clot lysis activity was subjected to regression and correlation to establish the IC_50_ value (half the maximum inhibitory concentration). The blood clotting inhibition method was used to determine the thrombolytic activity of the leaf, stem, and root extracts, yielding IC_50_ values of 173.7, 163.8, and 190.9 *μ*g/mL, respectively, with stem exhibiting the best thrombolytic activity when compared to the standard (100 *μ*L).

This study can also be supported by the work on *Camellia sinensis* where 90.34% clot lysis activity was found in leaves at a concentration of 800 *μ*g/mL [37].

### 3.3. Statistical Analysis

Finally, total phytochemicals (alkaloids, phenols, tannins, flavonoids, and proteins) were statistically examined for their effect on bioactivity (antioxidant, anti-inflammatory, and thrombolytic) in the study. For this, regression analysis and Pearson's correlation coefficient analysis were performed by using Microsoft Excel 2010.

In this analysis (Table 2), the correlation values (*r*) were calculated as 0.9 for phenolics, as 0.7 for flavonoids, as 0.51 for proteins, as 0.58 for alkaloids, and as 0.9 for tannins, which indicated a strong positive correlation for phenolics as well as tannins, whereas moderate positive correlation for flavonoids, proteins, and alkaloids.

This study was supported by the work on statistical analysis [38, 39] where correlations of phenols and flavonoids with antioxidant activity were discovered, which are compatible with the current study.

As a strong positive correlation between phenolics and tannins in bioassays was found, both were further statistically analyzed for checking the significance level.

Phenols and tannins of the plant were found to have (significance F) 0.043130 < 0.05 (phenol) and 0.04473 < 0.05 (tannin) *p* value; hence, it can be said that it has statistically significant correlations with antioxidant, anti-inflammatory, and thrombolytic effects, which accorded with the finding of a similar work [36].

## 4. Conclusion

For the first time, the phytochemical, antioxidant, anti-inflammatory, and thrombolytic potentials of *C. lanatum* were reported. According to the phytochemicals and bioactivities studied, the root of *C. lanatum* has the greatest antioxidant and anti-inflammatory potential, with the highest alkaloid and protein content, while the stem has the greatest thrombolytic activity, with the highest phenol, flavonoid, and tannin contents. Further research is recommended to fractionate and purify the extract to find the bioactive compounds responsible for the antioxidant, anti-inflammatory, and thrombolytic activities.

## Figures and Tables

**Table 1:** Qualitative phytochemical analysis of *C. lanatum*.

Test	Observation		
	Leaf	Stem	Root
Alkaloids			
Dragendroff's reagent	+++	+++	+++
Tannic acid	+++	+++	+++
Wagner	+++	++	+++
Hager	+++	+++	+++
Mayer	+++	+++	+++
Flavonoids	++	+++	+
Saponins	+	+	+
Tannins	+	+++	++
Phenols	++	+++	++
Terpenoids	+++	+++	+++
Steroids	+	++	++
Glycosides	+	+	+
Cardiac glycosides	+++	++	++
Quinines	+++	+++	++
Coumarins	+++	+	++
Proteins	+++	++	++
Resins	++	+	+

**Table 2:** Results of regression correlation analysis of the total content in bioassays.

Name	Protein	Flavonoid	Tannin	Phenol	Alkaloid
*R* ^2^	0.2632	0.6086	0.9745	0.9068	0.3378
*r*	0.51	0.78	0.9^*∗*^	0.9^*∗*^	0.58

*r*, correlation coefficient; *R*^2^, linear regression coefficient^∗^, Significant.

## Data Availability

The data used to support this study are included within the article and are available from the corresponding author upon request.
